# Cardiac Manifestations of Coronavirus Disease 2019 (COVID-19): A Comprehensive Review

**DOI:** 10.7759/cureus.8021

**Published:** 2020-05-08

**Authors:** Faryal Tahir, Taha Bin Arif, Jawad Ahmed, Farheen Malik, Muhammad Khalid

**Affiliations:** 1 Internal Medicine, Dow University of Health Sciences, Karachi, PAK; 2 Cardiology, Kansas City University of Medicine and Biosciences, Joplin, USA; 3 Cardiology, Ascension Via Christi Hospital, Pittsburg, USA

**Keywords:** acute cardiac injury, acute myocardial injury, cardiac injury, cardiac manifestations, covid-19, coronavirus disease (covid-19), covid-19 pandemic, sars-cov-2, pandemic, troponins

## Abstract

Since its origin in China, severe acute respiratory syndrome coronavirus 2 (SARS-CoV-2) infection has become a pandemic and spread to 209 countries. As coronavirus disease 2019 (COVID-19) is a very rapidly emerging disease, organ-specific studies related to it have been reported. Apart from respiratory findings, some studies have highlighted inflammatory consequences in the heart, kidney, and/or liver as well. Cardiac involvement in COVID-19 seems to be a result of an inflammatory storm in response to the infection. Moreover, direct viral invasion of cardiomyocytes, as well as a myocardial injury due to oxidative stress, may account for acute cardiac injury in COVID-19. Nevertheless, the mechanism of heart injury in COVID-19 is not clear yet. However, multiple studies that highlight the clinical features, laboratory findings, and prognosis of acute myocardial injury (AMI) in COVID-19-affected individuals have been published. In this review, we have summarized the findings of all those studies as well as the clinical features and management of cardiac injury discussed by some case reports.

## Introduction and background

Several epidemics and pandemics have plagued the earth since the beginning of time. The Plague of Justinian is believed to be the first pandemic in history, dating back to 541-750 AD, which was followed by the Great Bubonic Plague that killed over 125 million people [1]. Several different epidemics have been caused by beta-coronaviruses (Beta-CoV) in the last 20 years. The first epidemic was caused by severe acute respiratory syndrome coronavirus (SARS-CoV) from 2002 to 2003, followed by Middle East respiratory syndrome coronavirus (MERS-CoV) in 2012 [1].

Seven years after the MERS-CoV epidemic, an unknown pathogen infected the people in Wuhan, China, in December 2019. Patients reported to local hospitals with respiratory symptoms, and scientists eventually identified a new member of Beta-CoV as the causative agent for the disease [2]. On January 12, 2020, the World Health Organization (WHO) named the virus as the 2019-novel coronavirus (2019-nCoV). By January 20, cases of 2019-nCoV were reported from three other countries, namely Thailand, Japan, and South Korea. The genome was rapidly identified, and by January 30, this outbreak was declared a Public Health Emergency of International Concern; and the disease was named as coronavirus disease 2019 (COVID-19). On February 11, the Coronavirus Study Group of the International Committee on Taxonomy of Viruses named the new virus ‘SARS-CoV-2’. On March 11, the disease was declared a pandemic by the WHO [3].

Over the last three months, the disease has spread rapidly from 282 cases to over 2.1 million cases worldwide [3]. The basic reproduction number (R_0_) is an indicator of the transmissibility of a virus. It shows the expected number of new infections generated by an infected individual in a susceptible population. If R_0_ is more than 1, the number of new cases is likely to increase [4]. The mean R_0_ for SARS-CoV-2 is 3.28 (1.4-6.49), which is comparable to SARS, but the more widespread and rapid rise in the number of cases indicates higher transmissibility of SARS-CoV-2 [4].

As COVID-19 is a very rapidly emerging disease, new evidence and information are being reported daily. However, due to the emergence of such a large number of studies, disease- and organ-specific reviews are necessary to provide an updated and comprehensive summary of all literature for physicians who currently cannot spare their precious hours to go through vast online databases. Hence, we searched PubMed, Embase, Scopus, Google Scholar, ScienceDirect, Wiley, and coronavirus collections of all major publishing groups to identify literature related to cardiac involvement in COVID-19. In this review, we summarize the cardiac manifestations of COVID-19 and their prognoses.

## Review

Origin and epidemiology of COVID-19

In December 2019, people in Wuhan, China, started visiting local hospitals with pneumonia-like symptoms due to unknown causes. Many of the index cases’ history linked them to exposure to the Huanan Seafood Wholesale Market. China notified the WHO about the outbreak on December 31, 2019, and closed the Huanan market on January 1, 2020, for cleaning and disinfection [3]. On January 7, 2020, scientists were able to isolate and identify the sequence of 2019-nCoV. All genome sequences from different patients were almost identical, indicating the recent emergence of disease in humans [5].

**Figure 1:**
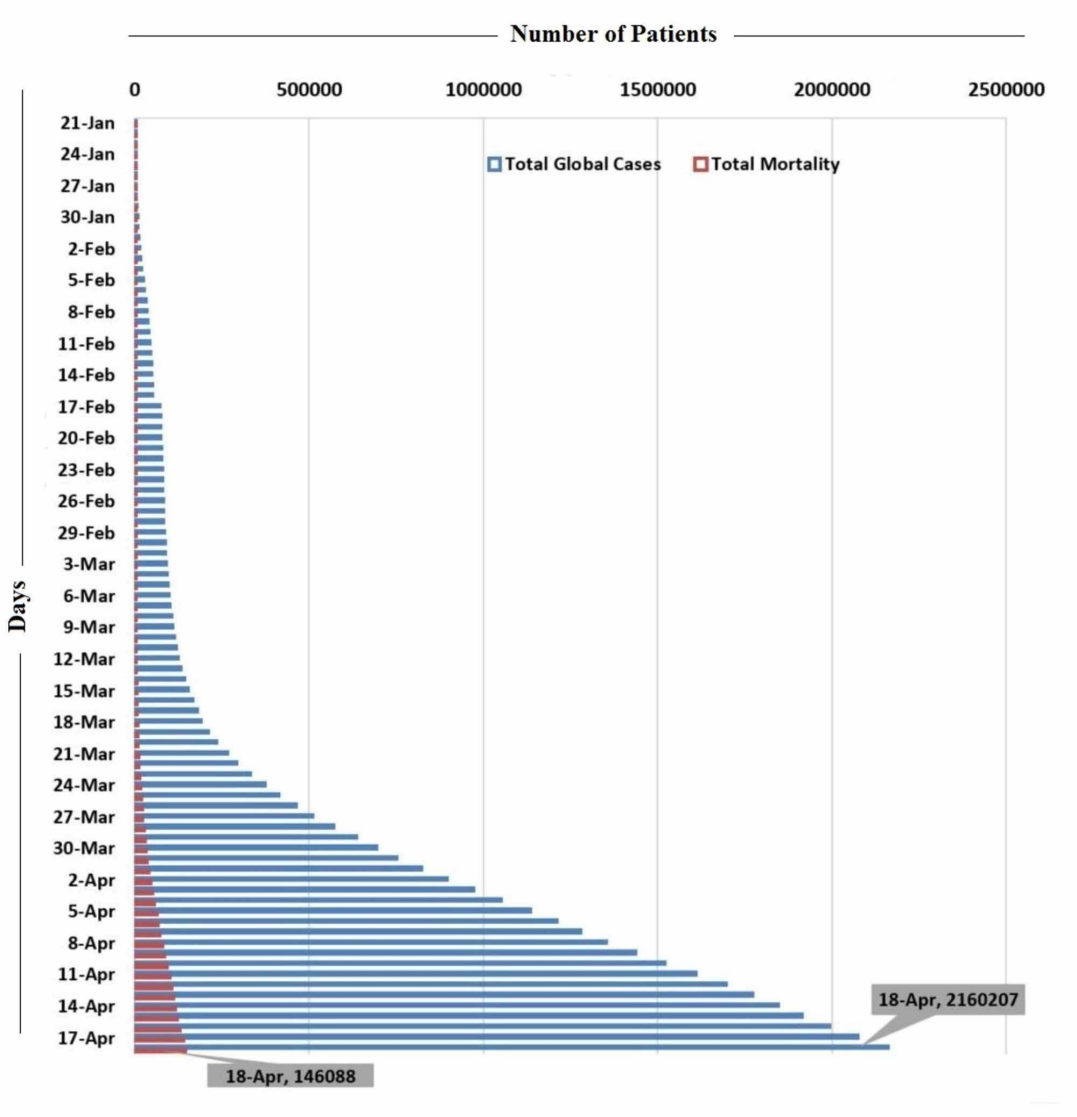
Representation of the number of COVID-19 cases and deaths globally from January 21 to April 18, 2020 The X-axis shows the number of patients and Y-axis shows the days COVID-19: coronavirus disease 2019

Since its origin in China, the SARS-CoV-2 infection has become a pandemic and spread to 209 countries. According to the latest WHO COVID-19 situation report (April 18, 2020), 2,160,207 cases and a total of 146,088 deaths have been reported so far. An overwhelming number of 6,710 new deaths were reported in the last 24 hours. The three countries with the highest number of cases (till April 18, 2020) are the United States, (665,330), Spain (188,068), and Italy (172,434) [3]. An increase in percentage mortality over time has been observed in the WHO COVID-19 status reports from January 21 to April 18, 2020 (Figure 1) [3].

Structure of SARS-CoV-2

Coronaviruses (CoVs) are a broad family of viruses that primarily affect the respiratory system. Four genera of CoV have been identified: alpha, beta, gamma, and delta. SARS-CoV-19, a ribonucleic acid (RNA) virus, belongs to subgenus Sarbecovirus of the genus Beta-CoV. A total of six other CoVs that can infect humans have also been identified. Other widely known members of the CoV family that were identified earlier include SARS-CoV and the MERS-CoV, both belonging to the beta genus and capable of causing life-threatening respiratory infections. The other four are human coronavirus (HCoV)-229E, HCoV-NL63, HCoV-HKU1, and HCoV-OC43, which cause milder disease [2,5,6].

The gross structure of SARS-CoV-2 identified in patients in Wuhan appeared spherical with some degree of pleomorphism on electron micrographs [2]. The diameters of viral particles were between 60 to 140 nm and the size of spikes was about 9-12 nm, which appeared like a crown [2]. The strain of SARS-CoV-2 is 29.9 kb [7]. Open reading frames (ORFs) vary in number from 6-11 in the CoV genome. The first ORF has two-thirds of viral RNA, which encodes for non-structural proteins and translates two polyproteins (pp1a and pp1b), while the structural and accessory proteins are encoded by other ORFs. The four essential structural proteins include (1) spike (S) glycoprotein, (2) nucleocapsid (N) protein, (3) matrix protein, and (4) envelope (E) protein [8-10].

SARS-CoV and MERS-CoV have genomes of 27.9 kb and 30.1 kb, respectively [11]. The SARS-CoV-2 is more closely related to two SARS-like CoVs derived from bats (bat-SL-CoVZC45 and bat-SL-CoVZXC21), with up to 88% similarity in identity, but distant from SARS-CoV and MERS-CoV (identity 79% and 50% respectively) [2,5]. This evidence suggests possible origin from bats [5].

Reservoir, host, and transmission

Based on its origin and spread in the Wuhan market, where there was an abundance of all sorts of animals, it is highly possible that COVID-19 has a zoonotic origin and it transmitted to humans through an intermediate host [10]. Identification of SARS-like CoVs in bats, which were similar to SARS-CoV-2, shows the possibility that bats can be a reservoir host for its progenitor [12]. It also indicates that mammals are likely to be a link between humans and COVID-19 [13]. Based on recent research, there are two species of snake that could also be a possible reservoir for SARS-CoV-2 [14]. However, no study has yet found any substantial evidence of a reservoir of SARS-CoV-2 other than birds and mammals. Human-to-human transmission of SARS-CoV-2 is possible and accounts for the majority of cases now [3,10,14].

SARS-CoV-2 is highly infective and transmission of the disease is through direct contact with an affected individual or through respiratory droplets produced by coughing or sneezing. There is recent evidence of possible vertical transmission from a mother to her newborn [15]. A recent study by Luo et al. has also shown that the virus is unlikely to be weakened in high temperatures and humidity and cluster transmission is possible even at conditions that were earlier thought to be unsuitable for viral transmission [16].

The higher infectivity of SARS-CoV-2 can also be understood through its higher viability in the air as well as on different surfaces [17]. SARS-CoV-2 can remain viable in air for more than three hours, and on plastic and steel for more than 72 hours. On copper and cardboard, the viability of SARS-CoV-2 is less than four hours and 24 hours, respectively [17]. This high viability time period on different surfaces can lead to indirect transmission of SARS-CoV-2 to healthy individuals if they come into contact with objects used on COVID-19 patients, such as stethoscope or thermometer [18]. There have been no cases of fecal-oral transmission to date, and airborne transmission is possible in situations where there is aerosol production such as bronchoscopy, open suctioning, nebulization, tracheostomy, intubation, or cardiopulmonary resuscitation [18-20]. Aerosol generation from the fecal matter of affected individuals, such as by flushing toilets or treatment of excreta in rural areas, may cause viral spread [21].

Pathogenesis and clinical presentation

SARS-CoV-2 uses angiotensin-converting enzyme 2 receptor (ACE2-R) for entry into the cells. S protein of the virus binds to ACE2-R. This binding causes a conformational change in S protein and leads to fusion of viral envelope with the host cell membrane, and SARS-CoV-2 RNA is released into the host cell. Genome RNA is translated into polyproteins (pp1a and pp1b), which are further broken into proteinases. Other viral proteins are produced by the translation of subgenomic messenger RNA (mRNA). In the endoplasmic reticulum (ER) and Golgi apparatus, the viral genome and proteins are assembled into virions. The newly formed virions are transferred to the cell membrane and released out of the cell [22].

ACE2 is found in the lower respiratory tract in humans, and the virus can be transmitted to the airway directly by respiratory droplets or touching of the nose after touching an infected surface [18,22]. Initial clinical presentation of the disease is usually asymptomatic or mild with symptoms such as cough, sore throat, rhinorrhea, headache, fever, shortness of breath (SOB), nausea, vomiting, diarrhea, and fatigue. In some patients, the disease can progress to pneumonia, respiratory failure, acute cardiac injury (ACI), multi-organ failure (MOF), and ultimately death [23,24].

A recently published study by Huang et al. reported detailed symptoms of 41 patients affected by COVID-19 [24]. Symptoms at onset commonly included fever (98%), cough (76%), and myalgia/fatigue (44%). Less common presenting symptoms were the production of sputum (28%), headache (8%), hemoptysis (5%), and diarrhea (3%). As the illness progressed, dyspnea, leukopenia, and lymphopenia developed in 55%, 25%, and 63% of the patients, respectively. All admitted patients developed pneumonia with bilateral pulmonary involvement (multiple consolidations and bilateral ground-glass opacity) visible on CT scan. A total of 15% of the admitted patients expired. Complications developed by patients included acute respiratory distress syndrome (ARDS) (29%), RNAaemia (15%), ACI (12%), secondary infection (10%), and complications requiring intensive care unit (ICU) admissions (32%) [24].

Level of inflammatory chemokines and cytokines such as interleukin (IL) 1-β, IL1RA, IL7, IL8, IL9, IL10, basic fibroblast growth factor 2 (bFGF2), granulocyte-colony stimulating factor (GCSF), granulocyte-macrophage colony-stimulating factor (GM-CSF), interferon-gamma (IFNγ), interferon-inducible protein 10 (IP10), monocyte chemoattractant protein-1 (MCP1), macrophage inflammatory proteins (MIP) 1α, MIP1β, platelet-derived growth factor subunit B (PDGFB), tumor necrosis factor-alpha (TNFα), and vascular endothelial growth factor A (VEGFA) were elevated in both ICU and non-ICU patients [24]. Pro-inflammatory cytokines, namely IL2, IL7, IL10, GCSF, IP10, MCP1, MIP1A, and TNFα, were only elevated in ICU patients, suggesting a possible association with increased disease severity [24].

Cardiac involvement in COVID-19

**Figure 2:**
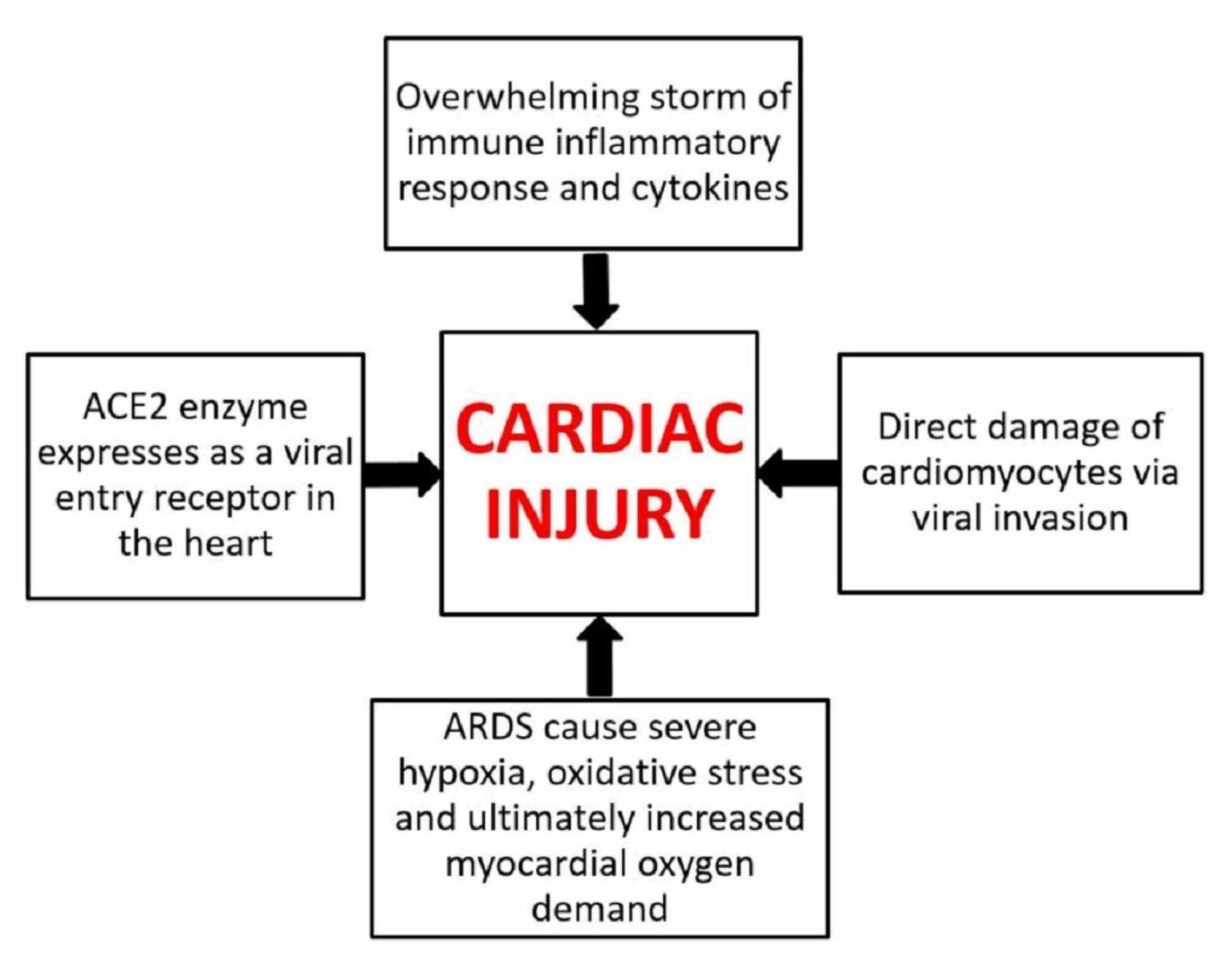
Mechanism of cardiac injury in COVID-19 ACE2: angiotensin-converting enzyme 2; ARDS: acute respiratory distress syndrome; COVID-19: coronavirus disease 2019

COVID-19 can lead to cardiac involvement and injury via the following possible mechanisms: (1) indirect injury due to increased cytokines and immune-inflammatory response, (2) direct invasion of cardiomyocytes by SARS-CoV-2, and (3) respiratory damage from the virus causing hypoxia leading to oxidative stress and injury to cardiomyocytes [25-27]. These mechanisms are graphically represented in Figure 2, which is adapted from [27].

Along with the presence of ACE2 in the respiratory tract, it is also found in the heart, and SARS-CoV-2 can use ACE2 to enter the cells [27,28]. The mechanism of heart injury in COVID-19 is still not entirely clear, i.e., it is unclear as to whether binding of SARS-CoV-2 alters the expression of ACE2 or causes abnormality in the regulation of the renin-angiotensin-aldosterone system (RAAS) [27]. However, multiple studies analyzing acute myocardial injury (AMI) in COVID-19-affected individuals have been published. AMI was detected by high levels of serum C-reactive protein (CRP), creatine kinase (CK), and troponins. Affected individuals often had a history of chronic diseases such as diabetes, chronic obstructive pulmonary disease (COPD), hypertension, and coronary artery disease (CAD). AMI was also associated with a longer hospital stay [24,29-43]. Critically ill patients had a higher likelihood of myocardial injury, which was associated with poor outcomes and an increased risk of in-hospital mortality [32,37,39,41].

Discussion of studies

Multiple studies that highlight the clinical features, laboratory findings, and prognosis of AMI in COVID-19-affected individuals have been published so far. These studies are discussed below.

In a single-center retrospective case series, Wang et al. delineated the epidemiological and clinical characteristics of novel coronavirus-infected pneumonia (NCIP) among 138 consecutively hospitalized patients at Zhongnan Hospital, Wuhan [29]. Epidemiological, demographic, clinical, laboratory, radiological, and treatment data were collected from January 1 to 28, 2020. The authors then compared the outcomes of critically ill and non-critically ill patients. Among all the patients, 54.3% (n=75) were male with a median age of 56 years. Comorbidities were found in 46.4% (n=64) of the patients, among whom cardiovascular disease (CVD) was appreciated in 14.5% (n=20). Among all the ICU-bound patients (n=36), 25% (n=9) were reported to have cardiovascular comorbidity. The median heart rate (HR) of all patients and those in the ICU was found to be 88 beats per minute (bpm) and 89 bpm, respectively. Similarly, the mean arterial pressure (MAP) of all patients and patients bound to ICU was recorded as 90 mmHg and 91 mmHg, respectively. ACI was appreciated in 7.2% (n=10), 22.2% (n=8), and 2% (n=2) in all, ICU bound, and non-ICU-bound (n=102) patients, respectively (p<0.001). Furthermore, arrhythmia was found in 16.7% (n=23), 44.4% (n=16), and 6.9% (n=7) in all, ICU-bound, and non-ICU-bound patients, respectively (p<0.001). To recapitulate, the authors established a mortality rate of 4.3% [29].

Huang et al. investigated the clinical features of patients infected with 2019-nCoV in Wuhan, China [24]. Out of a total 41 hospital-admitted patients identified as having laboratory-confirmed COVID-19, the majority (n=30, 73%) were men and less than half had underlying comorbidities such as diabetes (n=8, 20%), hypertension (n=6, 15%), and CVD (n=6, 15%). The median age was 49 years. ACI (n=5, 12%) was the third most common complication in these patients after ARDS and RNAemia. Out of 13 patients admitted to ICU, four (31%) had an ACI and five died. The study concluded that severe respiratory illness with 2019n-CoV infection with deteriorating complications was associated with ICU admission and a higher mortality rate [24].

Zhang et al. retrospectively evaluated the clinical characteristics of 82 hospitalized patients who died of COVID-19 from January 11 to February 10, 2020, in the Renmin Hospital, Wuhan [30]. In this study, the majority of the deceased patients were male (65.9%, n=54), with a higher mortality rate found among older individuals. Significantly, 80.5% (n=66) of non-survivors were >60 years of age. A major bulk (76.8%, n=62) of the deceased individuals had some existing comorbidity, and among them, 20.7% (n=17) had heart disease. Cardiac damage was found in 89% (n=73) of patients, while cardiac failure (CF) (14.6%, n=12) was the third most common cause of death after acute respiratory failure (ARF) (69.5%, n=57) and sepsis syndrome/MOF (28%, n=23). Along with other abnormal laboratory findings, levels of CRP (>10 U/L), cardiac troponin T (TnT, >0.04 pg/ml), CK (>200 U/L), myoglobin (>110 μg/L), lactate dehydrogenase (LDH, >250 U/L) and creatine kinase myocardial band (CK-MB, >5 ng/ml) were found to be much higher in 100% (n=58), 86.7% (n=52), 34.7% (n=25), 60% (n=42), 93% (n=68), and 30% (n=21) of the deceased cases, respectively. The authors concluded that either the virus itself or the storm of released cytokines in its response might have aggravated damage to vital organs, including the heart [30].

In a retrospective, single-center study of medical records, Hui et al. investigated the correlation between clinical features and ACI among patients affected with COVID-2019 pneumonia [31]. In this study, records of 41 consecutive cases of COVID-19 (19 males and 22 females) from Beijing Youan Hospital in China between January 21 and February 03, 2020, were studied, including two deaths. In view of the 6th guidance provided by the National Health Commission of China, 4.88% (n=2), 78.05% (n=32), 9.75% (n=4), and 7.32% (n=3) of the patients were labeled as light, mild, severe, and critical cases, respectively. The median age of the patients was found to be 47 years, and adult males were found more prone to develop a severe infection. Underlying comorbidities such as hypertension, CAD, diabetes mellitus (DM) type 2, and tumor were found in 24.4% (n=10) of the patients, while severe and critical cases were notably substantial in the cardiac-related chronic disease group. Moreover, as compared to the normal value of troponin I (TnI), a 40-fold increase in its peak value and a higher CRP level (191 mg/L) was recorded among critical patients, which translated into a higher risk of cardiac injury. Atrial fibrillation (AFib) with HR inclining up to 160 bpm was recognized in 4.88% (n=2) of patients; all of them were under critical category and eventually died. Reduced epicardial adipose tissue (EAT) density was shown on the CT scans of severe (-96.08 HU) and critical (-98.77 HU) cases, indicating cardiac inflammation. The authors concluded that patients under light and mild categories rarely exhibited cardiac injury, whereas it was found more commonly among severe and critical patients, where tachycardia, elevated levels of TnI, and low EAT density were regarded as the main risk factors. To intercept fatality among COVID-19 patients, the authors suggested close monitoring of the cardiac functioning of all patients, especially the severe and critical ones, and to seek possible interventions for patients evincing features of abnormal cardiac injury [31].

Wu et al. conducted a single-center retrospective cohort study to explore whether heart injury occurs in COVID-19 and if it aggravates mortality later [32]. Among 188 patients with COVID-19, the mean age was 51.9 years (range: 21-83 years) and 119 (63.3%) were male. Older patients and patients with comorbidity (especially hypertension) tended to have increased high-sensitivity troponin I (hs-TnI) levels on admission, and a high mortality rate was associated with high hs-TnI on admission (≥6.126 pg/mL). Further, it was noticed that high hs-TnI levels were associated with increased inflammatory levels [neutrophils, IL-6, CRP, procalcitonin (PCT)] and decreased immune levels [lymphocytes, monocytes, cluster of differentiation (CD)4+, and CD8+ T cells]. Raised CK-MB levels tended to occur only in male patients and current smokers and were associated with higher mortality, increased inflammatory levels, and decreased lymphocytes. Similarly, increased LDH and α-hydroxybutyrate dehydrogenase (α-HBDH) tended to occur in older and hypertensive patients and were associated with higher mortality rates, increased inflammatory, and decreased immune levels. Subsequently, hs-TnI (p=0.05) and LDH levels (p=0.022) on admission day were negatively correlated with survival days. Hence, it was concluded that heart injury signs arise in COVID-19, especially in older patients, hypertensives, and male patients with current smoking habits. Elevated levels of heart injury indicators are associated with higher mortality and shorter survival days. The authors suggested that COVID-19 might attack the heart by inducing an inflammatory storm and patients with signs of heart injury must be identified primarily and carefully treated by cardiologists [32].

To investigate the clinical characteristics and eventual prognosis of COVID-19 patients with already existing CVD, Peng et al. retrospectively analyzed 112 patients who visited the Union Hospital (Wuhan) from January 20 to February 15, 2020 [33]. Depending on the clinical severity of the disease, all the patients were grouped into two categories: critical (n=16) and general (n=96). In comparison with the general group, lymphocyte count [0.74×10^9^/L (0.34×10^9^-0.94×10^9^/L)] was found to be extremely low in the critical group, whereas, in the same group, CRP [106.98 mg/L (81.57-135.76 mg/L)], PCT [0.20 μg/L (0.15-0.48 μg/L)], and body mass index (BMI) [25.5 kg/m2 (23.0-27.5 kg/m2)] were found to be much higher. A further assortment of patients into groups of survivors and non-survivors showed higher BMI (>25 kg/m2) and lactic acid levels [1.70 mmol/L (1.30-3.00 mmol/L)] among the non-survivors, whereas the oxygen index [130 (102-415)] was found to be much lower in the same category. Keeping in view either the critical and general groups or the survivor and non-survivor groups, medication with ACE inhibitor and angiotensin receptor blocker (ARB) did not impact the morbidity and mortality related to COVID-19 in patients with any preexisting cardiac disease. Hence, the authors concluded that a higher risk of mortality existed among COVID-19 patients with already existing cardiac disease/s where fulminant inflammation, lactic acid accumulation, and thrombotic events are regarded as the main exacerbating factors [33].

A cross-sectional study by Chen et al. evaluated the cardiovascular damage in patients with COVID-19 [34]. A total of 150 COVID-19 cases were included from Tongji Hospital (Wuhan) and the patients were divided into groups of mild cases (n=126) and critical care cases (n=24). Age, hypersensitive CRP (hs-CRP), and serum creatinine levels of the patients were observed to be higher in critical care cases than in mild cases (all p<0.05). Critical care patients had a higher prevalence of males, elevated N-terminal pro-B-type natriuretic peptide (NT-proBNP) and cardiac troponin I (cTnI), hypertension, and coronary heart disease (CHD) (all p<0.05). Elevated cTnI [odds ratio (OR): 26.909, 95% CI: 4.086-177.226, p=0.001] and CHD (OR: 16.609, 95% CI: 2.288-120.577, p=0.005) were found to be the independent risk factors of critical disease status according to multivariate logistic regression analysis. The authors concluded that COVID-19 could significantly affect heart function and lead to myocardial injury. The two crucial independent determinants of clinical disease status in patients with COVID-19 were the past medical history of CHD and an increased level of cTnI [34].

Xu et al. investigated the clinical characteristics and risk factors of AMI in COVID-19 patients [35]. A total of 53 consecutive laboratory-confirmed and hospitalized COVID-19 patients were included in the study. Out of 42 (79.25%) patients with cardiac complications, the majority (n=30) had elevated cardiac enzymes followed by diastolic dysfunction (n=20), tachycardia (n=15), electrocardiography abnormalities (n=11), and AMI (n=6). All patients with AMI were aged >60 years, and five of them also had two or more comorbidities (hypertension, diabetes, CVD, and COPD). Furthermore, it was noticed that the severity of novel coronavirus pneumonia (NCP) was higher in these patients than in patients with non-definite AMI (p<0.001). Three of these AMI patients died while two remained hospitalized in the ICU. COVID-19 patients with AMI had higher mean systolic pressure than other groups (p<0.001). According to multivariate analysis, the major risk factor for cardiac abnormalities in COVID-19 patients were elevated CRP levels (p<0.001, OR: 1.186; 95% CI%: 1.047-1.344), NCP severity (p<0.001, OR: 2.896; 95% CI: 1.266-6.621), and underlying comorbidities (p<0.001, OR: 4.336; 95% CI: 1.11-38.55). The occurrence of hypertension was higher in the AMI group (n=4, 66.67%). Similarly, CVD was more frequently found in AMI patients (n=4, 66.67%), all of whom had a history of CAD with one having prior coronary artery bypass grafting (CABG). Other comorbidities like diabetes and COPD were found in all patients with a significant occurrence in AMI patients (diabetes: n=2; COPD: n=2) compared to other groups. About 30 COVID-19 patients exhibited elevated cardiac markers; out of them, AMI and non-definite AMI patients had higher cardiac markers than other groups (p<0.05). D-dimer levels on admission were also elevated in the AMI patients (median=1.65 µg/ml) and patients with abnormal cardiac markers (median=0.79 µg/ml), and were subsequently higher than those in patients with normal cardiac markers (median=0.55 µg/ml; p<0.001). Among 24 patients exhibiting echo abnormalities, a higher frequency was found in AMI patients, manifesting as left ventricular (LV) wall thickening and diastolic dysfunction, left atrial enlargement, LV enlargement, and mitral, aortic and triple valve regurgitation [35].

Liu et al. conducted a single-center retrospective study to analyze the association of cardiovascular manifestations with in-hospital outcomes of COVID-19 cases [36]. Case details of 41 consecutive hospitalized health staff with confirmed COVID-19 were collected at the Central Hospital of Wuhan, China. The mean age of the population was 39.1 ± 9.2 years. About 24 (58.5%) cases had cardiovascular manifestation (CVM). Patients with CVM had comparatively lower baseline lymphocyte count (p<0.001), had more incidence of positive nucleic acid detection of throat swab (p=0.011) and received more oxygen support (p<0.001). Similarly, the rate of in-hospital effects was significantly higher in the CVM group (p=0.001). Multivariate logistic regression analysis indicated that the coexistence of CVM and NCP was not independently associated with in-hospital adverse effects (OR: 2.23, 95% CI: 0.24-20.27, p=0.478). However, a tendency of significance was noticed in baseline lymphocyte count between the two groups in the model (OR: 0.15, 95% CI: 0.02-1.26, p=0.081) [36].

He et al. analyzed the clinical characteristics of severe or critically ill patients with COVID-19 and evaluated the impact of the complicated myocardial injury on the prognosis of these patients [37]. Fifty-four patients who met the criteria of severe or critical conditions of COVID-19 were included in this retrospective study. The median age of patients was 68; 24 (44.4%) patients had hypertension, 13 (24.1%) had diabetes, eight (14.8%) had CHD, and three (5.6%) had previous cerebral infarction. It was noticed that 24 (44.4%) patients were complicated with myocardial injury and 26 (48.1%) patients died during the hospital stay. Patients with myocardial injury had higher in-hospital mortality rates than those without myocardial injury (n=14, 60.9% with myocardial injury vs. n=8, 25.8% without myocardial injury, p=0.013). Moreover, patients with significant myocardial injury exhibited significantly higher levels of CRP (153.6 ng/L vs. 49.8 ng/L) and NT-proBNP (852.0 ng/L vs. 197.0 ng/L) than patients without myocardial injury (all p<0.01). This study postulated that severe or critically ill COVID-19 patients have a higher prevalence of myocardial injury, and these patients face a significantly higher risk of in-hospital mortality. Hence, it is vital to monitor and manage the myocardial injury during hospitalization for severe or critically ill COVID-19 patients [37].

In another retrospective comparison of the clinical features of recovered versus deceased patients with COVID-19, Deng et al. collected data from two hospitals in Wuhan [38]. Out of the total enrolled COVID-19 patients, 109 died during hospitalization, whereas 116 recovered. The median age was higher in the deceased group as compared to the recovered group (69 years vs. 40 years, p< 0.001). Levels of creatinine (89.00 μmol/L vs. 65.00 μmol/L, p< 0.001) and CRP (109.25 mg/L vs. 3.22 mg/L, p < 0.001) were found to be significantly higher in the deceased group versus the recovered one. Complications were recorded to be higher among the deceased group in which ACI (59.6% vs. 0.8%, p< 0.001) was found to be much higher as compared to the recovered group [38].

To investigate the epidemiological and clinical features of patients infected with COVID-19 and cardiac injury, Liu et al. collected and analyzed data of medical records from January 10 to February 24, 2020 [39]. Among 291 patients from Guangzhou Eighth People’s Hospital, 5.2% (n=15) had a cardiac injury. Patients with cardiac injury were comparatively older than those without injury; the median age was found to be 65 years. There was a male predominance among cardiac injury patients, as 73.3% (n=11/15) of them were male. Hypertension (46.6%, n=7) and CHD (20%, n=3) were the most predominant comorbidities among individuals presenting with a cardiac injury. Moreover, common symptoms of COVID-19 like fever (73.3%, n=11), cough (46.7%, n=7), headache or fatigue (33.3%, n=5), and dyspnea (26.6%, n=4) without any complaints of chest pain and palpitations were appreciated in this group. Patients who had cardiac injury showed relatively higher systolic blood pressure (BP) (132 mmHg vs. 124 mmHg), TnI (0.07 ug/L vs. 0.003 ug/L), and BNP (245.5 pg/mL vs. 18.5 pg/mL) levels when compared with those without cardiac injury. Nevertheless, the levels of CRP were found to be raised among all patients with cardiac injury (n=15/15). Among all the enrolled patients, ARDS (20%, n=3) and severe pneumonia (73.3%, n=11) were reported more commonly in cases with a cardiac injury, which complicated the disease. The authors concluded that cardiac injury can be commonly found among patients with COVID-19, where it worsens the clinical outcomes and, hence, should be regarded as a potential prognostic risk indicator [39].

Zhou et al. studied the clinical characteristics of myocardial injury in patients with COVID-19 [40]. A total of 34 patients with COVID-19 were included in this study who were further divided into two groups: patients with severe COVID-19 (n=26) and those with very severe COVID-19 (n=8). The median age was 63 years in the severe COVID-19 group and 67 years in the very severe group. The authors noted significantly increased cTnI (46.8 ng/L vs. 4.8 ng/L), CK (199 U/L vs. 88 U/L), α-HBDH (453 U/L vs. 245 U/L), and LDH (513 U/L vs. 287 U/L) in the very severe group as compared to the severe group. The percentage of very severe patients with elevated cTnI levels was markedly higher as all eight patients with very severe disease exhibited increased cTnI while only one patient with severe disease had raised cTnI levels (p<0.001). This study concluded that patients with very severe COVID-19 have a higher percentage of increased cTnI levels and their mortality rate can be improved by protecting them from myocardial injury [40].

To explore the association between cardiac injury and mortality in patients with COVID-19, Shi et al. conducted a single-center cohort study at Renmin Hospital of Wuhan University, China [41]. They included a total of 416 hospitalized patients with COVID-19 in the final analysis, with a median age of 64 years. About 82 patients (19.7%) were declared to have a cardiac injury. In comparison with patients without cardiac injury, these individuals were older (p<0.001), had more comorbidities such as hypertension (p<0.001), had higher leukocyte counts (median: 9,400) and higher levels of CRP (median: 10.2), PCT (median: 0.27), CK-MB (median: 3.2), myohemoglobin (median: 128), hs-TnI (median: 0.19), NT-proBNP (median: 1,689), aspartate aminotransferase (AST, median: 40), and creatinine (median: 1.15), and had a higher proportion of multiple mottling and ground-glass opacity in radiographic findings (64.6%). Patients with cardiac injury required a greater proportion of non-invasive mechanical ventilation (p<0.001) or invasive mechanical ventilation (p<0.001). Similarly, complications like ARDS (p<0.001), acute kidney injury (AKI, p<0.001), electrolyte disturbances (p=0.003), hypoproteinemia (p=0.01), and coagulation disorders (p=0.02) were higher in patients with a cardiac injury. A higher mortality rate (p<0.001) was noticed in patients with cardiac injury (51.2%) than those without cardiac injury (4.5%). Additionally, patients with cardiac injury were at a higher risk of death, both during the time from onset of symptoms (HR: 4.26, 95% CI: 1.92-9.49) and from admission to endpoint (HR: 3.41, 95% CI: 1.62-7.16) according to Cox regression model. The study concluded that cardiac injury is a prevalent condition among hospitalized patients with COVID-19 in Wuhan, China, and it is associated with a higher risk of in-hospital mortality [41].

In order to depict the clinical characteristics of 113 deceased patients owing to COVID-19 infection, Chen et al. retrospectively evaluated a case series among a cohort of 799 confirmed cases of COVID-19 admitted to Tongji Hospital from January 13 to February 12, 2020 [42]. In comparison with the recovered cases, deceased patients were mostly male (73%, n=83) with a median age of 68 years. Among all those who died, chronic hypertension (48%, n=54) and other cardiovascular abnormalities (14%, n=16) were the predominant comorbidities. Vital signs revealed higher median systolic BP (137 mm Hg), arterial pressure (≥140 mm Hg), and HR (101.0 bpm) among deceased patients. Apart from other abnormal laboratory findings, considerably higher levels of CRP, CK, LDH, cTnI (40.8 pg/mL), and NT-proBNP (800 pg/mL) were appreciated among the deceased patients. Moreover, eight deceased patients exhibited more than 1,000 pg/mL cTnI whereas two of them had more than 10,000 pg/mL. The disease was more frequently complicated among the deceased patients with the occurrence of ACI (n=72/94) and HF (n=41/83) in 77% and 49% of the individuals, respectively. It was also observed that individuals with cardiovascular comorbidity were more susceptible to the development of cardiac complications. However, cardiac injury and HF were still more common among deceased patients as compared to the recovered ones, regardless of any history of cardiac disease. The authors concluded that ACI and HF were among the most common complications that affected the prognosis of critically ill patients [42].

**Figure 3:**
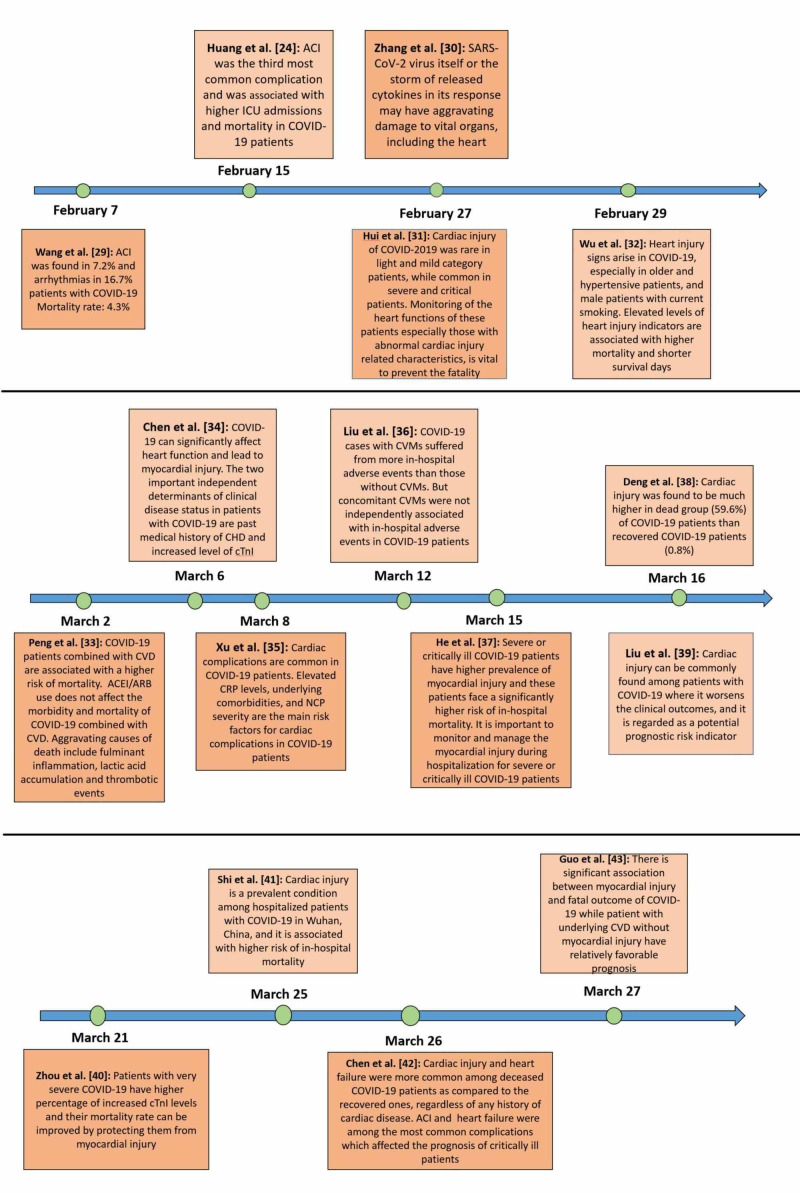
Flowchart with a summary of all the included studies ACI: acute cardiac injury; ICU: intensive care unit; COVID-19: coronavirus disease 2019; CHD: coronary heart disease; cTnI: cardiac troponin I; CVMs: cardiovascular manifestations; CVD: cardiovascular disease; ACEI: angiotensin-converting enzyme inhibitors; ARBS: angiotensin receptor blockers; CRP: C-reactive protein; NCP: novel coronavirus pneumonia

To evaluate the association of underlying CVD and myocardial injury with fatal outcomes in patients with COVID-19, Guo et al. conducted a retrospective single-center study [43]. A total of 187 patients with confirmed COVID-19 were selected, and their demographic data, laboratory findings, comorbidities, and treatments were analyzed regardless of elevation in TnT levels. Overall, 66 (35.3%) patients had underlying CVD manifested as hypertension, CHD, and cardiomyopathy, while 52 (27.8%) patients exhibited myocardial injury as indicated by elevated TnT levels. Patients with CVD and elevated TnT levels had a higher mortality rate (69.44%) than other groups, i.e., patients without underlying CVD and normal TnT (7.62%), patients with underlying CVD and normal TnT (13.33%), and patients without underlying CVD but elevated TnT (37.5%). Similarly, patients with underlying CVD were more likely to present with elevated TnT than those without CVD (54.5% vs. 13.2%). Plasma TnT levels depicted a significantly positive linear correlation with plasma hs-CRP (β=0.530, p<0.001) and NT-proBNP levels (β=0.613, p<0.001). Similarly, plasma TnT and NT-proBNP levels elevated significantly during hospitalization (median: 0.307 for plasma TnT; median: 1,902 for plasma NT-proBNB) and indicated impending death (median: 0.141 for serum TnT; median: 5,375 for serum NT-proBNP) as compared with admission values (median: 0.0355 for serum TnT; median: 796.90 for serum NT-proBNP) in the patients who died. However, no significant changes in TnT and NT-proBNP were observed in survivors. Patients with elevated TnT developed frequent malignant arrhythmias and required more glucocorticoids (71.2% vs. 51.1%) and mechanical ventilation (59.6% vs. 10.4%) as compared to those with normal TnT levels. The mortality rate of patients with and without the usage of ACEI or ARB was 36.8% and 26.5%, respectively. These observations concluded that there is a significant association between myocardial injury and fatal outcome of COVID-19 while the patients with underlying CVD without myocardial injury have a relatively favorable prognosis [43]. Figure 3 provides a summarized flowchart of all studies discussed above [24, 29-43].

Discussion of case reports

A few case reports have also depicted the distinctive involvement of the cardiovascular system in COVID-19 infection. The first case to report the CVM of COVID-19 was presented by Zeng et al. [44]. The article highlighted fulminant myocarditis as a complication of COVID-19 in a 66-year-old patient with no known comorbidity, who presented with fever, cough, dyspnea, and chest tightness. During hospitalization, myocardial enzymes showed elevated TnI (11.37 g/L), myoglobin (390.97 ng/mL), and NT-proBNP (22,600 pg/ml). Electrocardiogram (ECG) showed sinus tachycardia with no ST-segment elevation. However, an enlarged LV (61 mm), diffuse myocardial dyskinesia along with low left ventricular ejection fraction (LVEF, 32%), pulmonary hypertension (44 mmHg), and decreased inferior vena cava (IVC) collapse rate were noticed on bedside echocardiography. Severe pneumonia, ARDS, fulminant myocarditis, and multiple organ dysfunction syndrome (MODS) were considered as differentials. There was no improvement in the patient’s condition with supportive treatment. The ventricular septum thickened to 14 mm, and IL-6 increased by 272.40 pg/ml. Extracorporeal membrane oxygenation (ECMO) was used to reduce the cardiopulmonary burden, which showed a reduction in TnI (0.10 g/L), NT-proBNP (750 pg/ml), and IL-6 (7.63 pg/ml). The LVEF gradually recovered to 68%, and the LV and wall regained their normal thickness after treatment [44].

Inciardi et al. highlighted cardiac involvement as a complication of COVID-19 without symptoms and signs of interstitial pneumonia in a 53-year-old patient [45]. ECG of this patient showed diffuse ST elevation. Elevated hs-TnT and NT-proBNP levels were also detected. Findings on chest radiography were normal. Cardiac MRI showed increased wall thickness with diffuse biventricular hypokinesis, predominantly in the apical segments, and severe LV dysfunction (LVEF of 35%). Short tau inversion recovery (STIR) and T2-mapping sequences depicted marked biventricular myocardial interstitial edema and a diffuse late gadolinium enhancement involving the entire biventricular wall. A circumferential pericardial effusion, especially around the right cardiac chambers, was also observed. These findings were consistent with acute myopericarditis, and the patient was treated with dobutamine, antiviral drugs, steroids, chloroquine, and medical treatment for HF, with progressive clinical and instrumental stabilization [45]. Similarly, bilateral pericardial and pleural effusion as a complication of COVID-19 infection has been reported by Albarello et al. [46].

2019-nCoV infection may also involve other organs like heart, vessels, liver, and kidney, as demonstrated by Yao et al. [47]. The minimal invasive autopsies in three COVID-19 cases revealed degeneration and necrosis of parenchymal cells, the formation of hyaline thrombus in small vessels, and pathological changes of chronic diseases in other organs and tissues, while no evidence of coronavirus infection was observed in these organs. The authors suggested conducting further studies to investigate the mechanism of underlying extra-pulmonary pathological changes of this disease [47]. Further, Cui et al. highlighted multiple organ damage and rapid disease changes in a 55-day-old infant diagnosed with COVID-19 pneumonia [48]. Laboratory investigations on the day of admission revealed mildly abnormal myocardial zymogens with altered liver function tests (LFTs). With the deterioration of the patient’s condition, an increase in TnI (0.025 μg/L) was noticed, which indicated myocardial injury. Intravenous sodium creatine phosphate was added to her regimen for the protection of her heart. Later, the myocardial zymogen and liver function improved with supportive treatment, and the patient was discharged [48]. A summary of all the important findings of the aforementioned case reports is given in Table 1.

**Table 1:** Cardiac manifestations of COVID-19: summary of reported cases *Only abstract is available in English COVID-19: coronavirus disease 2019; CT: computed tomography; 2019-nCoV: 2019-novel coronavirus

Author	Patient's age, years	Patient's gender	Main findings
Zeng et al. [44]	63	Male	COVID-19 patients may develop severe cardiac complications (e.g., myocarditis and heart failure). The first case to report fulminant myocarditis as a complication of COVID-19
Inciardi et al. [45]	53	Female	Acute myocardial inflammation in a COVID-19 patient who recovered from the influenza-like syndrome and developed fatigue and signs and symptoms of heart failure a week after upper respiratory tract symptoms
Albarello et al. [46]	67	Male	COVID-19 infection can be complicated with bilateral pleural and pericardial effusion as noticed on subsequent chest CT scan of the patient
Yao et al.* [47]	-	-	The lung is the primary site of infection by 2019-nCoV. However, it can cause damage in other organs (heart, vessels, liver, kidneys) as demonstrated by the autopsies of three COVID-19 patients
Cui et al. [48]	55	Female	Children with COVID-19 can present with multiple organ damage. Myocardial injury is one of the complications associated with COVID-19

## Conclusions

We can conclude that ACI is common in COVID-19, especially among adult males, where it is regarded as a potential prognostic risk indicator. Higher levels of cytokines, IL, cardiac injury markers such as troponin and CK, and CRP are usually appreciated in these patients. Consequently, myocarditis, ventricular dysfunction with or without pericardial effusion, and subsequently, HF cumulatively increase the mortality rate. Hence, careful monitoring and immediate management plan to avoid or treat cardiac injuries are vital to achieving a decline in the number of deaths due to HF secondary to COVID-19.
